# Glycosylation of H4 influenza strains with pandemic potential and susceptibilities to lung surfactant SP-D

**DOI:** 10.3389/fmolb.2023.1207670

**Published:** 2023-06-13

**Authors:** Lisa M. Parsons, Olga Zoueva, Gabrielle Grubbs, Ewan Plant, Ewa Jankowska, Yijia Xie, Hao Song, George F. Gao, Zhiping Ye, Surender Khurana, John F. Cipollo

**Affiliations:** ^1^ Food and Drug Administration, Center for Biologics Evaluation and Research, Division of Bacterial, Parasitic and Allergenic Products, Silver Spring, MD, United States; ^2^ Food and Drug Administration, Center for Biologics Evaluation and Research, Division of Viral Products, Silver Spring, MD, United States; ^3^ Research Network of Immunity and Health (RNIH), Beijing Institutes of Life Science, Chinese Academy of Sciences, Beijing, China

**Keywords:** glycoproteomics, collectin, reassortment, pathogenicity, sialic acid, swine, glycopeptides

## Abstract

We recently reported that members of group 1 influenza A virus (IAV) containing H2, H5, H6, and H11 hemagglutinins (HAs) are resistant to lung surfactant protein D (SP-D). H3 viruses, members of group 2 IAV, have high affinity for SP-D, which depends on the presence of high-mannose glycans at glycosite N165 on the head of HA. The low affinity of SP-D for the group 1 viruses is due to the presence of complex glycans at an analogous glycosite on the head of HA, and replacement with high-mannose glycan at this site evoked strong interaction with SP-D. Thus, if members of group 1 IAV were to make the zoonotic leap to humans, the pathogenicity of such strains could be problematic since SP-D, as a first-line innate immunity factor in respiratory tissues, could be ineffective as demonstrated *in vitro*. Here, we extend these studies to group 2 H4 viruses that are representative of those with specificity for avian or swine sialyl receptors, i.e., those with receptor-binding sites with either Q226 and G228 for avian or recent Q226L and G228S mutations that facilitate swine receptor specificity. The latter have increased pathogenicity potential in humans due to a switch from avian sialylα2,3 to sialylα2,6 glycan receptor preference. A better understanding of the potential action of SP-D against these strains will provide important information regarding the pandemic risk of such strains. Our glycomics and *in vitro* analyses of four H4 HAs reveal SP-D-favorable glycosylation patterns. Therefore, susceptibilities to this first-line innate immunity defense respiratory surfactant against such H4 viruses are high and align with H3 HA glycosylation.

## Introduction

The prevalence and subtype distribution of the low-pathogenicity avian influenza virus (LPAIV) differ across bird taxa. A crucial factor in the epidemiology of these viruses is the ability to transmit between and within different host taxa (van Dijk et al., 2018). Although LPAIV infections are often asymptomatic, these viruses can serve as progenitors for reassortment and recombination for the eventual rise of high-pathogenicity avian influenza virus (HPAIV), which can lead to significant disease burden in wild and domestic fowl and swine. The AIV may also transmit to humans. In the 1990s, HPAIV H5N1 spread through 60 countries, causing devastating outbreaks of avian influenza with over 800 cases of human infections and 450 deaths (World Health Organization, 2020). A novel LPAIV H7N9, first discovered in China in 2013, caused severe infection in humans with nearly 1,600 laboratory-confirmed cases reported and 39% mortality as of April 2017 (Gao et al., 2013; Liu et al., 2014). Four years after this outbreak, H7N9, with mutations shown to increase virulence in chickens (HPAIV), was recovered from two human patients in China (Zhang et al., 2017).

H1, H2, and H3 IAVs have adapted to humans, and pandemics of each are hallmarks of these zoonotic transformations including the 1918 H1N1 Spanish flu, 1957 H2N2 Asian flu, 1968 H3N2 Hong Kong flu (Capua and Alexander, 2002), and 2009 H1N1 swine flu (Shapshak et al., 2011). Other IAVs have caused sporadic cases in humans such as H5N1 and H7N9 described previously. In the laboratory setting, four mutations in H5N1 have been identified that facilitate infection of ferret and human hosts (Herfst et al., 2012; Lu et al., 2013; Zhang et al., 2013; Shi et al., 2014). Two of these mutations, Q226L and G228S (H3 numbering), are in the receptor-binding domain of HA, leading to enhanced binding to sialylα2,6-capped receptors, which are abundant in swine (Bateman et al., 2010), ferret (Jia et al., 2014), and human respiratory tissues (Jia et al., 2014). Although no such mutations have been found in the H5N1 viruses in nature, this situation clearly shows their potential for adaptation to alternative hosts, including humans, and indeed, analogous mutations have been detected in the H4 viruses covered herein.

Live poultry markets (LPMs) are considered a major source of IAV dissemination, reassortment, and interspecies transfers (Liu et al., 2003; Bi et al., 2016). The IAV H4 subtype, which infects domestic ducks, is known to circulate in LPMs in China (Kawaoka et al., 1988) (Wu et al., 2014a) and has been isolated from swine suffering from pneumonia in Canada (Karasin et al., 2000). Transmission to swine populations in southeastern China and in humans has also been reported (Ninomiya et al., 2002) (Bateman et al., 2008; Kayali et al., 2011). A recent phylogenetic analysis of 3,020 cloacal swabs from apparently healthy ducks resulted in the identification of 107 influenza strains with eight HA subtypes and six NA subtypes (Deng et al., 2013). A second phylogenetic analysis of 3,210 cloacal swabs resulted in the identification of 109 strains with 10 HA subtypes (H1, H2, H3, H4, H5, H6, H7, H9, H10, and H11) and eight NA subtypes (N1, N2, N3, N4, N6, N7, N8, and N9) for a total of 21 IAV subtype combinations. In both studies, the most abundant HA subtype was H4, and based on a comparison of all genes, the reassortment (gene exchange) between the different subtypes was widespread. The findings showed that H4 and H3 viruses have reassorted and that H4 HAs have evolved considerably at the receptor-binding site (RBS) (Deng et al., 2013). A recent phylogenetic tree of H4 viruses was analyzed by Quan et al. (2018).

Among key RBS residues, Q226 and G228 (H3 numbering) are associated with an avian sialylα2,3 receptor preference. However, H4 IAV isolates from Canadian swine have been reported to contain L226 and S228, which is predicted to shift the RBS to a sialylα2,6 preference, both a swine and human tissue receptor (Wu et al., 2015). We have previously reported that this prediction is borne out (Song et al., 2017), as X-ray crystallographic analysis revealed that the Q226L and G228S mutations reduce sialylα2,3 ligand contacts, whilst increasing sialylα2,6 contacts in the RBS. Surface plasmon resonance and tissue binding analyses targeting sialylα2,3 or sialylα2,6 receptors supported the structural findings. The abilities of H4 to resort with IAV H3, which is prevalent in humans, to evolve to recognize swine and human receptors and to infect humans, albeit sporadically to date, all suggest that H4 has the potential to infect humans in a pandemic event.

Are these influenza H4 viruses susceptible to lung surfactant protein D (SP-D), a primary innate defense surfactant collectin? Indeed, a key glycosylation site in the head of H3 required for interaction with SP-D co-aligns with that of H4 align as we will demonstrate herein, and this site is centered at N165 (H3 numbering). Our previous work demonstrated that this glycosylation site can be either primarily high-mannose or primarily complex N-glycan subtype groups according to the IAV group, at least in part, and that subtype is the key to the SP-D interaction.

IAV HAs can be classified into two broad groups based on phylogenetic analysis. Group 1 contains H1, H2, H5, H6, H8, H9, H11, H12, H13, H16, H17, and H18, while group 2 contains H3, H4, H7, H10, H14, and H15 (Wu et al., 2014b; Allen and Ross, 2018). We recently reported the susceptibilities of group 1 LPAIV to lung SP-D. H2, H5, H6, and H11 whole virus and recombinant HAs were investigated (Parsons et al., 2020). Glycoproteomics analysis of these HAs revealed that they all contained the complex H3 relative N165 (“N165”) glycosylation site. H3 HAs from group 2, on the other hand, contain almost exclusively high-mannose *N-*glycans at the N165 site in the globular head region, which is required for SP-D recognition of H3 HA and subsequent elimination from the lung (Hartshorn et al., 1994; Hartshorn, 2010). In our work, the “N165” glycosite of H2, H5, H6, and H11 HAs was located more forward on their respective resident beta strand compared to that of H3 viruses. This situation results in fewer intra- and inter-subunit contacts than the H3 N165 resident *N-*glycan core. This orientation of the glycosite predicts more exposure to solvents in the group 1 LPAIV strains studied, as well as higher mobility and more exposure to ER and Golgi glycosylation machinery. The predicted outcome is more processed glycans at that site yielding not high-mannose glycans but complex ones, which are not receptors for SP-D. This prediction was confirmed by our glycoproteomics analysis (Parsons et al., 2020). The analyzed group 1 LPAIV HAs had almost completely complex glycan at “N165.” Experiments with recombinant versions of SP-D also demonstrated that compared to HA of H3 strains (group 2), HAs of all group 1 LPAIV strains tested (H2, H5, H6, and H11) were not ligands for SP-D (Parsons et al., 2020). Constructs yielding group 1 HAs with only high-mannose glycan at “N165” imparted SP-D sensitivity, demonstrating that the SP-D interaction can be imparted with high-mannose glycan at “N165” in group 1 IAV. The prediction would be that if the LPAIV infected humans through gain of function adaptation, the resulting IAV would potentially be highly pathogenic as it would not be acted upon efficiently by SP-D in the lung. As SP-D is a primary innate immunity factor (Reading et al., 2007), this situation could be detrimental. Indeed, HPAIV H5 infections in humans have occurred and demonstrate high mortality (Govorkova et al., 2005; de Jong et al., 2006; Chen et al., 2007), and those H3 strains that have lost key high-mannose glycans in the head of HA become more pathogenic in model systems (Hartshorn et al., 2008).

Our glycoproteomics work with the group 1 LPAIV and group 2 H3 HAs revealed a high correlation between “N165” location, glycan subtype, susceptibility to surfactant SP-D, and the presence or absence of key residues in the 220 loop, especially W222 or its alternative R222 (H3 numbering). Upon analysis of representative H4 sequences (Supplementary Figures S1, S2) containing the avian Q226/G228 and swine L226/S228 (H3 numbering as per the work of Bateman et al. (2008), Wu et al. (2015), and Song et al. (2017)) receptor region amino acid patterns, which dictate avian and swine receptor preferences, respectively (Parsons et al., 2020), we noted that, in all cases, the H4 HA N165 placement aligned with that of H3 HA. Unlike H2, H5, H6, and H11, the corollary glycosite “N165” in H4 is recessed back in its resident beta strand like H3, thus less solvent exposed. However, some H4 strains, such as the duck strain studied here, have L222, whilst swine and teal H4 IAVs, also studied here, contain W222. Therefore, as the position on the beta strand and the presence or absence of key amino acid residues influence the glycosylation in this region, the question is what is the glycan subtype in the H4 HAs? In addition to the demonstrated shift in receptor preference dictated by the evolution of Q226L and G228S mutations, interactions of the N165 glycan with key residues, including either L222 or W222, may dictate the glycan subtype and, therefore, sensitivity to SP-D. The status of RBS preference for sialylα2,6 glycans is considered a strong marker for pathogenicity. Here, we investigate site N165 status as a second possible pathogenicity marker based on predicted interactions with SP-D and test the susceptibility *in vitro* with SP-D constructs.

## Materials and methods

### Chemicals and reagents

HyperSep C18 and porous graphite carbon (PGC) cartridges with 100-mg bed weight were purchased from Thermo Fisher Scientific Inc. (Waltham, MA). TSKgel Amide-80 particles were purchased from Tosoh Bioscience LLC (Montgomeryville, PA). Sequencing-grade modified trypsin was purchased from Promega Corp. (Madison, WI). Peptide N-glycosidase F (PNGase F) was purchased from New England BioLabs Inc. (Ipswich, MA). Iodomethane, dimethyl sulfoxide (DMSO), sodium hydroxide beads, and other chemicals were purchased from Sigma-Aldrich (St. Louis, MO, USA); solvents were of high-performance liquid chromatography (HPLC) grade or higher. All other reagents were American Chemical Society (ACS) grade or higher.

### Strains

Four strains were studied. A/duck/Czechoslovakia/1956 (H4N6), A/blue-winged teal/Wisconsin/402/1983 (H4N6), A/blue-winged teal/Illinois/10OS1563/2010 (H4N6), and A/swine/Missouri/A01727926/2015 (H4N6) were acquired from the Center for Biologics Evaluation and Research (CBER) as egg-grown. The viruses were propagated in eggs and purified as described previously (An et al., 2013). Strains, growth conditions, and abbreviations are given in Table 1.

**TABLE 1:** H4 influenza strains and growth conditions analyzed in this study.

Sample	Strains	W222	Growth conditions
Duck	A/duck/Czechoslovakia/1956 (H4N6)	No	Egg
Teal83	A/blue-winged teal/Wisconsin/402/1983 (H4N6)	yes	Egg
Teal10	A/blue-winged teal/Illinois/10OS1563/2010 (H4N6)	yes	Egg
Sw15	A/swine/Missouri/A01727926/2015 (H4N6)	yes	Egg

### Whole-virus glycopeptide preparation

Of the total mass of each of the four egg-grown whole-virus samples, 2 mg was processed. Upon reconstitution, enough dry urea to equal 8 M in the existing volume was added, resulting in concentrations of about 6 M. Dithiothreitol (DTT) was added to reach 5 mM, and the samples were incubated for 3 h at 37°C. After cooling, iodoacetamide was added to a concentration of 15 mM, and the samples were stored in the dark at room temperature for 30 min. The reaction was quenched by increasing the concentration of DTT to a total of 30 mM. Using a 10-MWCO membrane, the ∼3 mL samples were dialyzed three times into 500 mL of 50 mM ammonium bicarbonate, pH 8.0 (ammonium bicarbonate (ABC) buffer). The samples were concentrated using 10-MWCO spin filters. The final amount of the modified protein was approximately 100 μg of proteins per sample as measured by Bearden’s assay. Trypsin was added at an enzyme:protein ratio of 1:35 (w/w), and the samples were incubated at 37°C overnight. After collecting LC/MS data on the trypsin-cleaved, HILIC-purified sample, ABC buffer (ammonium bicarbonate 50 mM; pH 8.0) was added to the remaining 12 μL of samples to reach a concentration of 50 mM, and chymotrypsin was added at an enzyme:protein ratio of 1:20. The sample was incubated at 37°C overnight, and data were collected the next day.

### N-glycan release

About 20 μg of each glycopeptide was dried and resuspended in 50 μL O^18^ water or normal water (sw15 only) with 50 mM ammonium bicarbonate, pH 8.0. The samples were heated for 10 minutes at 95°C to inactivate trypsin. Glycans were then released with 10 uUnits/μL of PNGase F overnight at 37°C.

### Purification of deglycosylated peptides and free glycans

Deglycosylated peptides were captured using C18 cartridges preconditioned with 1 mL ethanol and then 1 mL of water. Glycans were eluted with 3 mL of deionized water and peptides subsequently eluted with 60% acetonitrile (ACN)/0.1% tri-fluoroacetic acid (TFA), dried in a SpeedVac concentrator, and resuspended in 15 μL water for nanoLC-MS^E^ analysis.

The C18 free glycan eluates were further purified using a PGC column. The C18 eluates were reconstituted in 1% 1-butanol (v/v). The PGC column was prepared by sequential 1 mL washes of 100% ACN, 60% ACN/0.1% TFA, 30% ACN/0.1% TFA, and 0.1% TFA in water. The loaded columns were washed three times with 1 mL 0.1% TFA/water. Glycans were eluted with 1 mL 30% ACN/0.1% TFA and then 1 mL 60% ACN/0.1% TFA. The eluents were pooled and dried in glass vials by rotary evaporation.

### Enrichment of glycopeptides

Intact glycopeptides were enriched from the trypsin cleavage mixture with TSKgel Amide-80 hydrophilic interaction liquid (HILIC) resin as described previously (An and Cipollo, 2011). In brief, 200 μg of resin (400 μL of wet resin) in a 1 mL Supelco fritted column was washed with 1 mL of 0.1% TFA in water, followed by equilibration with 1 mL of 80% ACN with 0.1% TFA. Trypsin-treated samples were diluted with pure ACN and 0.1% TFA to make a final ACN concentration of 75% to avoid precipitation. The samples were loaded, and the run-through was re-applied to maximize capture. The columns were washed three times with 1 mL of 80% ACN/0.1% TFA, sequentially eluted with 1 mL 60% ACN/0.1% TFA and then 1 mL 40% ACN/0.1% TFA. The eluents were combined, dried by vacuum centrifugation, and then, resuspended in 25 μL MS-grade water for analysis by nanoLC-MS^E^.

### Permethylation of free N-glycans

The samples were permethylated following the protocol of Ciucanu and Kerek (1984) with modifications as described previously (Parsons et al., 2017).

### MALDI-TOF analysis of permethylated N-glycans

Permethylated glycans were spotted in triplicate on a hydrophobic surface 384 circle μFocus plate (Hudson Surface Technology) as follows: each spot was pre-treated with 1 μL DHB matrix solution (20 mg/mL 2.5-dihydroxybenzoic acid in 50% ACN/50% water with 1 mM sodium acetate) and air-dried. The permethylated glycans were resuspended in 50% ACN and mixed 1:1 (v/v) with the DHB solution on each spot and dried. Spotted surfaces were recrystallized at 50°C upon the addition of 1 μL of 100% ethanol. A total of 4,000 shots were collected and summed from each spot using a Bruker AutoFlex MALDI-ToF/ToF. A mass ladder of permethylated maltooligosaccharides was used as an external calibrant. Data were calibrated and smoothed, and the baseline was subtracted using FlexAnalysis 3.4 (Bruker Daltonics) and automatically assigned using AssignMALDI (Parsons and Cipollo, 2023). AssignMALDI uses a glycan library compiled from public glycan databases.

### NanoLC-MS^E^ analysis of glycopeptides and peptides

Approximately 2–4 μg of the prepared glycopeptide or deglycosylated peptide sample was injected three separate times onto a C18 column (BEH nanocolumn, 100 μm i.d. X 100 mm, and 1.7 μm particles; Waters Corporation). Parameters were as described previously (Parsons et al., 2017). Initial calibration of the Waters SYNAPT G2 HDMS system (Waters Corp., Milford, MA) was performed in the MS^2^ mode using glufibrinopeptide B in 50% ACN/0.1% TFA.

### Data analysis for peptide and glycopeptide information

NanoLC-MS^E^ data were processed using BiopharmaLynx 1.3x (Waters) and GLYMPS (in-house software (Parsons et al., 2017; An et al., 2019; Parsons et al., 2019; Parsons et al., 2020)). Settings for trypsin-digested peptides were 1 missed cleavage, fixed cysteine carbamidomethylation, variable methionine oxidation, and variable *N*-glycan modifications. After initial processing by BiopharmaLynx to assign fragment ions to the correct precursor ions, GLYMPS was used to automatically assign the spectra with a building-block glycan database as described previously (Parsons et al., 2017). Mass accuracy was set in GLYMPS to 30 ppm, and assignments were based on (1) the presence of a core fragment (peptide, peptide + HexNAc, peptide + HexNAc2, peptide + dHex1HexNAc2, or peptide + Hex1HexNAc2) (World Health Organization, 2020), (2) the presence of three or more assigned glycopeptide fragments (Gao et al., 2013), (3) the presence of three or more assigned peptide fragments (Liu et al., 2014), (4) the presence of the assignment in at least two out of three spectra (Zhang et al., 2017), and (5) the existence of the glycan in the GlycoSuiteDB.

### Molecular comparisons

The three-dimensional structures of HAs with PDB codes 4FNK (H3), 5XL1 (H4 duck), and 5XL2 (H4 swine) were aligned, and bonds were measured using CCP4MG (CCP4 Molecular Graphics).

### SP-D real-time kinetics assay using surface plasmon resonance

Dodecameric human SP-D neck and carbohydrate domains with D325A and R434V mutations (gift from Kevan Hartshorn) (Crouch et al., 2011a) were labeled with EZ-Link NHS-PEG4-Biotin (Thermo Fisher) following the manufacturer’s instructions. MALDI-TOF (Bruker Autoflex™ Speed) analysis in the linear positive mode using sinapinic acid (10 mg/mL in 50% acetonitrile) as the matrix verified at least one, and up to five lysines were labeled per monomer when the reaction was carried out with a label concentration 20 times that of the protein. The protein was mixed with 0.45 mL of phosphate-buffered saline (PBS) and concentrated three times using a 0.5 mL 10 KDa MWCO centrifugal filter (Amicron) to exchange the buffer, concentrate the protein, and remove excess labels.

Binding of different influenza viral strains to biotinylated SP-D glycan was analyzed at 25°C using a ProteOn surface plasmon resonance biosensor (Bio-Rad Labs). In some cases, the viruses were first digested using endoglycosidase F1. In brief, 0.03 U Endo F1 and 10 μL of 250 mM sodium acetate, pH 4.5, were added to approximately 25 μg of antigen with 33.5 μL of deionized water. The reaction mixtures were incubated at 37°C for 1 h and then dialyzed against PBS. Biotinylated SP-D was coupled to an NLC sensor chip at 350 resonance units (RUs) in the test flow cells. Three-fold serial dilutions (30-, 90-, and 270-fold) of freshly prepared influenza virus samples in PBS containing 10 μM neuraminidase inhibitors (oseltamivir and zanamivir) were injected at a flow rate of 50 μl/min (120-s contact time), followed by dissociation for 600 s. The flow was directed over a mock surface to which no protein was bound, followed by the SP-D-coupled surface. Responses from the SP-D surface were corrected for the response from the mock surface and for responses from a separate, buffer-only injection. Kinetic data analyses were performed to calculate the apparent affinity constant for the interaction between the influenza virus and SP-D using Bio-Rad ProteOn manager software (version 2.0.1). Viral particle counts all measured between 1 × 10^8^–2 x 10^9^ vp/mL.

## Results

### Protein sequence alignment analysis

Previously, we compared seasonal H3 HAs, from group 2, to H2, H5, H6, and H11 HAs from group 1 IAV (Parsons et al., 2020). As described earlier, herein, we discovered that the local placement, contacts, and orientation of glycosite “N165” had a dramatic effect on the N-glycan subtype present at the glycosite. Here, we compared the H3, H4, and other group 2 HA sequences (Supplementary Figures S1, S2). We found that the analogous “N165” glycosite of H4 (N162 in H4 numbering) aligns with the H3 position rather than with group 1 strains such as H5. This was the case for all four H4 HAs analyzed in this study (Supplementary Figure S2) and is also the case for the consensus sequence derived from all 1,117 non-redundant H4 sequences in the flu database (Supplementary Figure S1). As previously reported, the H3 HA N165 glycan interacts strongly with the neighboring subunit 220 loop. Multiple contacts between the inner glycan core and W222 were observed. We note that hereafter, numbering used is that of H3 to be consistent with previous publications describing the recent changes in the H4 RBS (Shi et al., 2016; Song et al., 2017). The two teal and the swine H4 strains studied here contain W222. However, the duck strain contains L222. Not unexpectedly, the swine sequence also contains L226 and S228, which have been shown to shift the RBS to a sialylα2,6 preference, while all the avian sequences have Q226 and G228 consistent with sialylα2,3 preference. Overall, the prediction would be that H4 HA with W222 would have a similar glycosylation subtype to H3 HA (high-mannose at “N165”), whereas those with L222 may or may not, depending on the intra- and intermolecular connectivities at the site. Strains used in this study are shown in Table 1.

### Overall glycosylation

**FIGURE 1:**
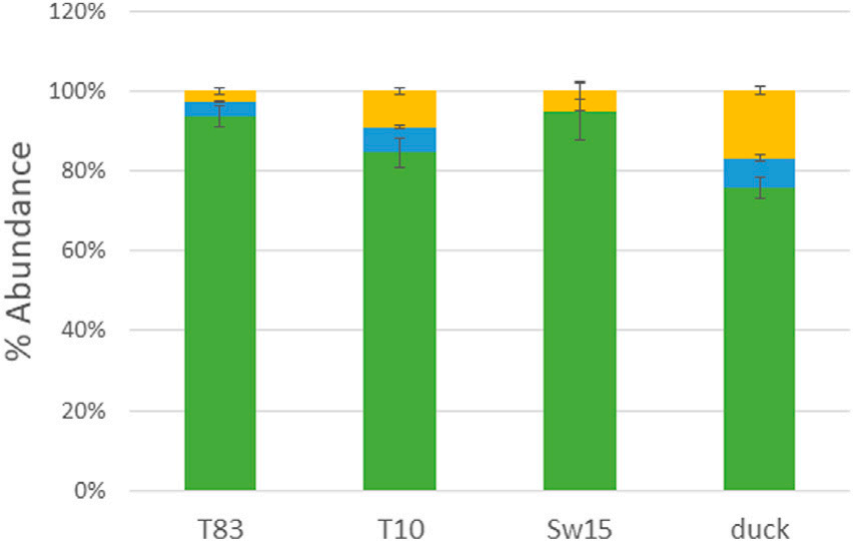
MALDI-TOF MS analysis of PNGase F-released glycans from H4N6 strains. Histogram representations are shown with green representing high-mannose, blue representing hybrid, and yellow representing complex glycans.

**FIGURE 2:**
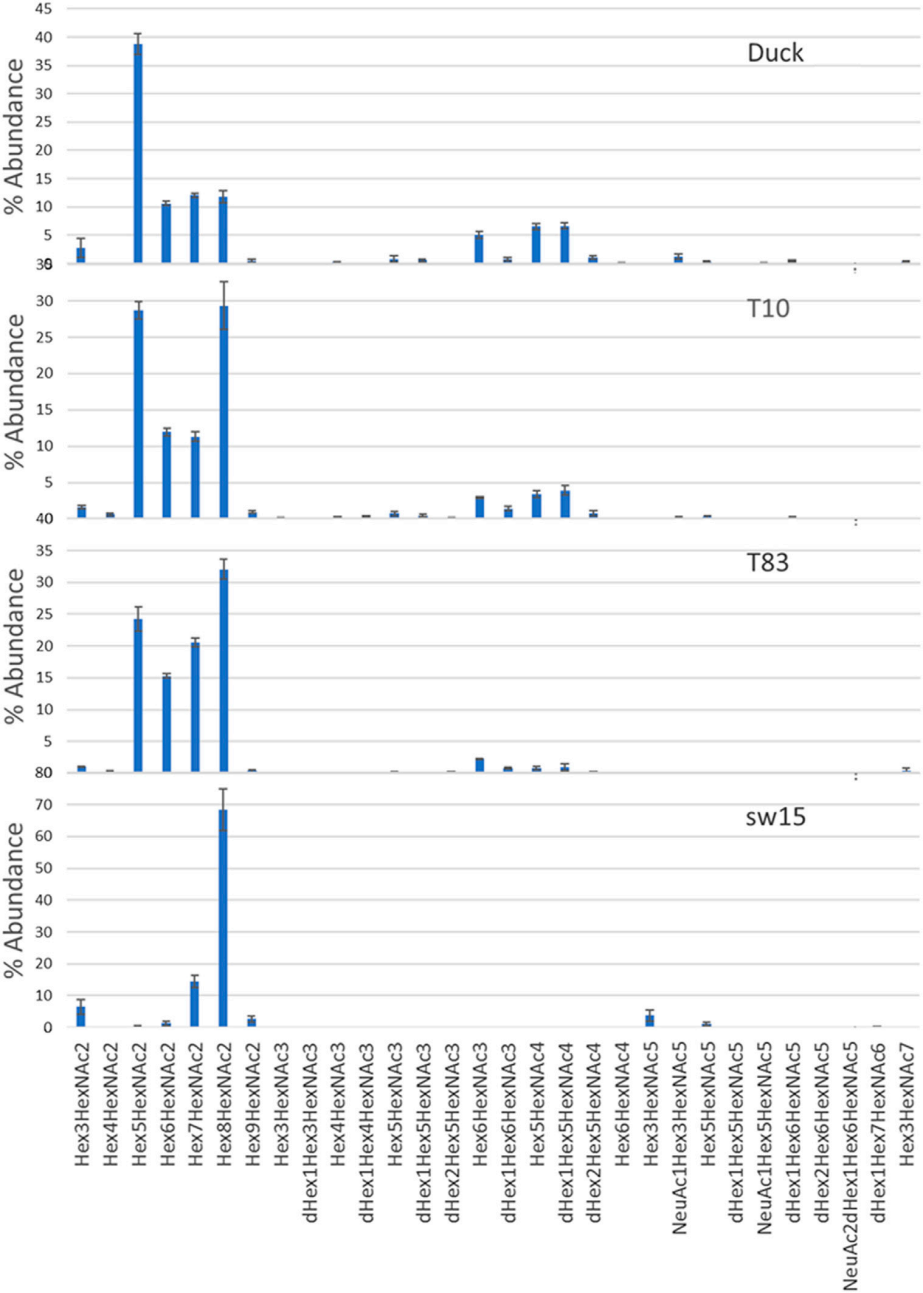
Detailed compositions of MALDI-TOF MS analysis of PNGase F-released glycans from H4N6 strains. A comparison of glycoforms grouped by the number of N-acetylhexosamines (HexNAcs2, HexNAcs3, or HexNAcs4+) is shown, which is reflective of complexity, where the former is primarily high mannose, the second is hybrid, and the last is complex glycans.

PNGase F-released glycans from whole viruses grown in eggs were permethylated and analyzed by MALDI-TOF MS. The overall glycoform distributions for the four HAs are shown in Figure 1. The figure shows the predominance of high-mannose glycans. Figure 2 shows a detailed comparison of glycoforms grouped by the number of *N-*acetylhexosamines (HexNAcs2, HexNAcs3, or HexNAcs4+), which is reflective of complexity. Those with HexNAc2 are associated with high-mannose glycans. Those with three HexNAcs are associated with hybrid or short complex ones, and those with more than three HexNAcs are associated with complex glycans. These associations are well-documented in the literature and accepted as such (Kornfeld and Kornfeld, 1985; Schachter, 2000; Parsons et al., 2019; Parsons et al., 2020; Shajahan et al., 2020). Although high-mannose glycans dominated the profiles, both hybrid and complex glycans were also present. It should be noted that the overall glycosylation of the whole-viral samples grown in eggs here represents all glycosylation present in the virus and not just that of HA. Glycosylation on other proteins such as neuraminidase will affect the overall abundance, although hemagglutinin is the most abundant glycoprotein present in the virus. Typically, HA glycans represent more than 85% of the released glycans as it is approximately 70% of viral protein and is highly glycosylated. See (cited) as an example.

### Occupancy

NetNGlyc is a publicly available online software application published in 2004 to predict the glycosylation sites in human proteins (Gupta et al., 2004). It was used in this study to identify glycosylation sequence and their potential for glycosylation. Five sites are predicted to be glycosylated in the four strains studied (Table 2). Sites N2 and N165 of the mature sequence are predicted to have the highest potential to be glycosylated, sites N18 and N481 are predicted to have low-to-intermediate potential, and site N294 is predicted to have the least.

**TABLE 2:** NetNGlyc predicted glycosylation.

Potential glycosylation sites		NetNGlyc predictions			
Position	Sequence	Duck	Sw15	Teal83	Teal10
2	NYTG	***^a^	***	***	***
18	NGTM	*	**	**	**
165	NLTK	***	***	***	***
294	NISR	*	*	*	*
481	NGTY	**	*	**	**

^a^ The higher the number of *, the higher the potential to be glycosylated.

### Glycopeptide analysis

**FIGURE 3:**
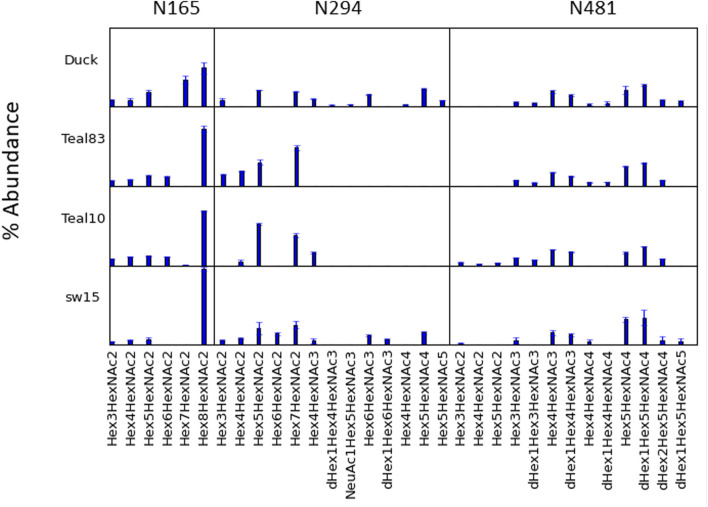
Glycosylation profile comparisons at each glycosylation site. Individual compositions at N165, N294, and N481 are shown for each of the four H4N6 strains examined. N165 is essentially only high mannose, whilst N294 may contain primarily high mannose or up to a mixture of high-mannose, hybrid, and complex types depending on the strain. N481 contains primarily hybrid and complex N-glycans.

**FIGURE 4:**
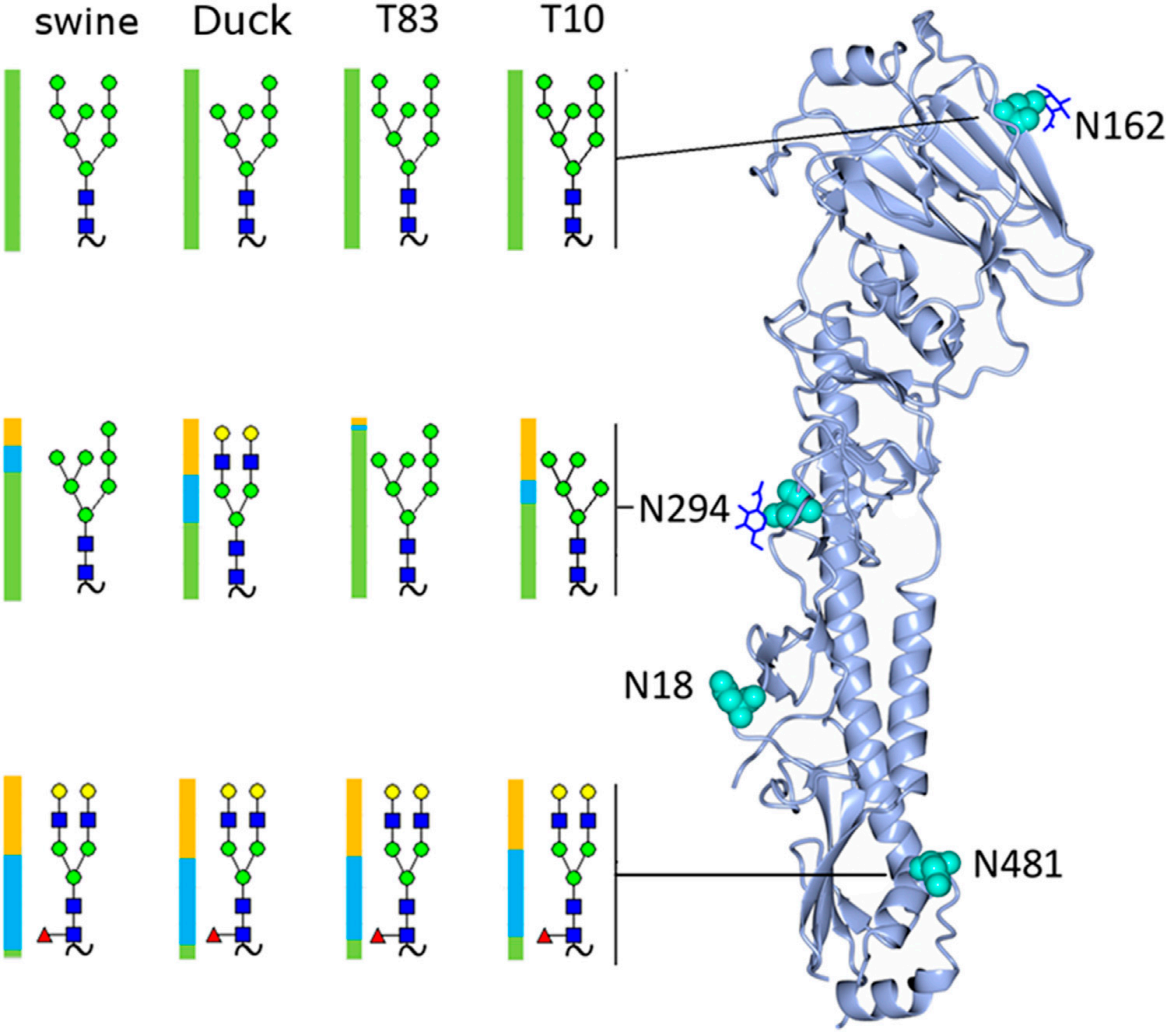
Rendering of the H4 monomer with the most abundant glycan detected at each site shown and the distribution of glycan subtypes shown with a colored bar where green represents high-mannose, blue hybrid, and yellow complex glycans.

LC/MS^E^ analysis of glycopeptides was performed on HILIC-enriched, trypsinized samples and on HILIC-enriched, trypsinized glycopeptides subsequently treated with chymotrypsin. The glycan heterogeneity and relative intensity at individual glycosites are shown in Figure 3. Only trace abundances of glycopeptides containing N2 or N18 were found in the Teal10, Teal83, or SW15 samples. While the signal intensity from the duck sequence at the N2 site was weak, multiple glycosyl and peptide fragments were found consistent with high-mannose glycans. This is likely because the glycopeptide was rather long and hydrophilic, thus resulting in lower ionization efficiency. The percent abundance of N165, N294, and N481 glycopeptide assignments found in all samples and a comparison are shown in Figure 4 and listed in Supplementary Table S1. The range of glycans, their abundance, and their glycan family subtype distributions at each site were similar across the egg-derived HAs. The N165 site in all four samples (duck, teal83, teal10, and sw15) was populated with only high-mannose forms with the majority being the Hex_8_HexNAc_2_ in all samples. Representative spectra are shown in Supplementary Figures S4–7. N294 glycosylation was mostly high-mannose in the two teal samples but a combination of high-mannose and HexNAc3-4-containing glycoforms in the duck and swine (SW15) virus samples. N481 glycan was mostly a hybrid and complex glycan across all four samples with very similar abundances.

A full list of assignments is given in Supplementary Tables S1A–D. The most abundant glycoform at each site in each sample is shown in Figure 4, along with its location on the hemagglutinin monomer. The samples contained predominantly high-mannose glycans on the head (site N165) and mid (site N294) locations.

### Molecular comparison of H3 and H4 HA crystal structures

**FIGURE 5:**
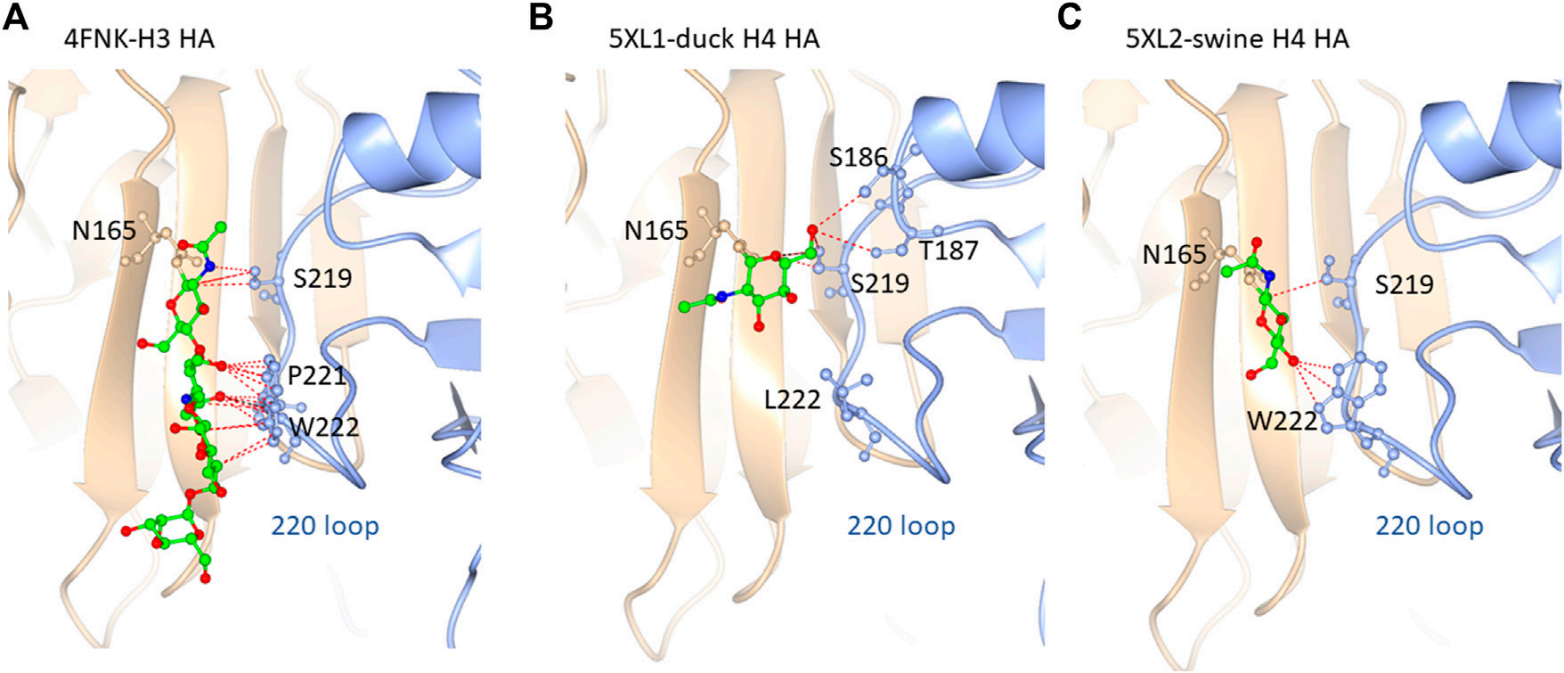
N165 glycosylation site for **(A)** H3 (FNK-H3 HA), **(B)** 5XL1-duck H4 HA, and **(C)** 5XL2-swine H4 HA. The resident beta sheet is shown in beige and the 220 look in blue. The glycan core orientation observed in the crystal structure is shown with predicted contacts. See Supplementary Figure S2 and test for details.

Based on our glycoproteomic mass spectrometry analysis, glycosite N165 is occupied exclusively by high-mannose glycans. Figure 5 shows the representative crystal structure projections showing the intramolecular contacts observed in and around glycosite N165 for H3 HA from A/Hong Kong/1/1968 (Figure 5A), H4 HA from A/duck/Czechoslovakia/1956 (Figure 5B), and H4 HA from A/swine/Missouri/A01727926/2015 (Figure 5C). A full list of the molecular contacts is given in Supplementary Table S2. PDB structures of the teal sequences are not available, but they are about 96% identical to the duck sequence and about 98% identical to the swine sequence, and both teal sequences have W222, similar to the swine sequence (see Supplementary Figure S2 for a sequence alignment). As previously reported, H3 HA N165 is more *N-*terminally located on the beta strand in a less solvent-exposed region than that of the group 1 viruses from our previous study, which included HAs, H2, H5, H6, and H11, where the corollary site is more C-terminal and solvent-exposed (Parsons et al., 2017; Parsons et al., 2020). The location of the H4 HA N165 glycosite is more similar to that of the H3 HAs. As seen in the H3 HA representation in Figure 5A, the inner core of the high-mannose glycan forms extensive contacts with the neighboring subunit 220 loop residues S219, P221, and W222. Most of these are between the second GlcNAc of the glycan and P221 and W222, with W222 aligning in a planar arrangement to the chitobiose (GlcNAc2) rings. Overall, 20 contacts were counted. Figure 5B shows the structure of the duck HA studied here. The H4 HA N165 glycosite is located in the analogous position on the beta sheet compared to H3 HA. However, the neighboring subunit 220 loop has leucine instead of tryptophan at position 222, and no contacts with this amino acid are observed with the N165 glycan core. There are also no contacts to P221. Intra-subunit contacts are observed between the aglycone-most GlcNAc (closest to the glycosidic linkage) and S186, T187, and S219. The rest of the glycan cannot be seen, suggesting that there are little to no contacts between the rings of the chitobiose unit and the 220 loop, unlike that in the H3 structure. Figure 5C shows the structure of the swine HA. N165 is again placed in the same position as that of H3 HA. In this case, W222 is present. However, unlike the H3 configuration, the chitobiose unit of the N165 glycan is not planarly oriented with W222, although several contacts are made between the O4 oxygen of the aglycone GlcNAc and the NE1 nitrogen, CE2, and CZ2 carbons of W222. Contact is also made between the GlcNAc C1 and S219 carbon CB. Supplementary Table S2 shows a list of contacts shown in Figure 5 and described herein.

In summary, the H4 HAs all contained exclusively high-mannose glycans at N165, and crystal structure data examination reveals the contacts and orientation of the H4 HA N165 glycosite that limits solvent exposure compared to group 1 HAs previously examined and similar to the group 2 H3 HA as shown here. The H4 N165 structural orientation likely limits exposure of the site to glycosylation processing machinery, thus limiting glycosylation subtypes to high-mannose glycan. Interestingly, the high-mannose glycans at site N165 on the duck H4 HA, which has L222 and fewer contacts with the 220 loop (Figure 5), had more Hex7HexNAc2 than the viruses with W222, which had predominantly Hex8HexNAc2 at N165 (Figure 3). Hex7HexNAc2 glycoforms are further along the glycan processing pathway than Hex8HexNAc2 glycoforms. It is tempting to speculate that the additional processing occurs due to the apparently less structured N165 in the duck H4 HA.

### SP-D binding characteristics of avian and human IAV HAs by surface plasmon resonance

We tested the relative binding preference of H4 duck (A/duck/Czechoslovakia/1956), T10 (A/Bwteal/IL/100S/1563/2010), T83 (A/Bwteal/Wisconsin/402/83), and SW15 (A/Swine/Mo/A017/27926/15) and control viruses A/Ny/470/2004 (H3N2): positive control, A/pintail/339/1987 (H3N8): positive control, and A/mallard/Ohio/249/1998 (H6N1): negative control. Recombinant human SP-D (rhSP-D) (dodecamer) was used in the study and was characterized previously (Hartshorn et al., 2000). SP-D was derivatized with biotin and coupled to NLC sensor chips. Three-fold serial dilutions (30-, 90-, and 270-fold) of freshly prepared influenza virus samples in PBS containing 10 μM neuraminidase inhibitors (oseltamivir and zanamivir) were analyzed. Interferograms are shown in Supplementary Figure S3, and calculated Kd values are shown in Table 3. H4 and H3 viruses had highly similar dissociation contents, consistent with similar binding properties to SP-D. All H4 viruses examined are ligands for recombinant SP-D and likely to be efficiently acted upon by endogenous SP-D. We extended our analysis to assess if reducing the high-mannose glycans selectively would reduce SP-D binding. Our previous unpublished and published studies (An et al., 2019) revealed that the conditions required for digestion of intact non-denatured HA resulted in the loss of detectable potency as tested using the single radial immunodiffusion (SRID) test. Furthermore, CD analysis revealed a change in the detected secondary structure after PNGase F digestion. Additionally, if the enzyme had worked, the release would have been indiscriminate for the subtype of N-glycan. However, digestion with Endo F1, F2, and F3 preserved CD patterns, and SRID detected potency. We used endoglycosidase F1, which exclusively targets high-mannose and hybrid glycans (Maley et al., 1989).

**TABLE 3:** Surface plasmon resonance analysis of IAV strains.

Strain	HA type	Kd (M)
A/blue-winged teal/Wisconsin/402/1983	H4	1.01E-07
A/duck/Czechoslovakia/1956	H4	1.75E-07
A/blue-winged teal/Illinois/100S1563/2010	H4	8.20E-08
A/swine/Missouri/A01727926/2015	H4	1.71E-07
A/NY/470/2004 (H3N2)	H3	1.21E-07
A/pintail/339/1987 (H3N8)	H3	5.26E-08
A/mallard/Ohio/249/1998 (H6N1)	H6	No binding

Although complete digestion cannot be expected under the mild and native-preserving conditions used and the recessed position of site N165, we did detect a reduction in SP-D binding comparable to that of H3, which is known to bind SP-D in a high-mannose N165 glycosylation site-dependent manner (Hartshorn et al., 1996; Hartshorn et al., 2008; Crouch et al., 2011b; Parsons et al., 2020) (Supplementary Table S3).

## Discussion

The domestic duck is positioned at the boundary between wild aquatic and terrestrial fowl. This situation is known to play an important role in the ecology of the IAV (Li et al., 2004; Huang et al., 2010; Huang et al., 2012; Kim et al., 2012). H4 influenza was initially isolated in the former in Czechoslovakia in 1956 and is now known to widely circulate in domestic fowl in Asia, Europe, and North America. This LPAIV has been known to resort with other influenza subtypes, and there is evidence, for instance, that H4 viruses have reassorted with H3 and H11 IAV (Teng et al., 2012; Deng et al., 2013). Transfer of H4 LPAIV from an avian host to domestic swine has been reported (Karasin et al., 2000). Reassorted H4 strains isolated from domestic fowl live poultry markets were shown to infect mice without prior adaptation (Wu et al., 2015). Furthermore, H4 IAV has crossed the species barrier to infect humans. This has been seen in Lebanese poultry workers as revealed through serologic antibody detection by microneutralization and RBC hemagglutination assays (Kayali et al., 2011). In 2000, H4 HAs from isolates of swine were shown to harbor mutations at amino acid residues 226 and 228, specifically, Q226L and G228S (Karasin et al., 2000). The G228S mutation alone imparted dual-receptor specificity such that both sialylα2,3 and sialylα2,6 receptors were preferred, explaining the alignment of receptor specificity with an abundant swine host ligand. Addition of the Q226L mutation refined the specificity more toward sialylα2,6 receptors, thus have more preference for the human sialyl ligand, which is more predominantly this linkage type (Bateman et al., 2008). As swine are an intermediary hosts between avian and human species, this situation is a cause for concern for human infection. The current work investigated a possible further pathogenic characteristic, i.e., the potential susceptibility of the LPAIV strains to lung SP-D, a primary innate immune system lectin prevalent in respiratory tissue and known to be a key factor in reducing influenza burden in the lung (Hartshorn et al., 1994; Watson et al., 2020).

A key factor in the interaction between SP-D and influenza virus is a specific glycosylation site on the globular head of HA. In the H3 viruses, this site is located at N165 and nearly exclusively harbors a high-mannose glycan. Extensive evidence in the literature shows that the high-mannose glycan interacts with specific regions in SP-D, whereby the Manα1,3- linked arm residues are key (Crouch et al., 2009; Crouch et al., 2011b). Previously, we investigated representatives of the avian LPAIV from H2, H5, H6, and H11 viruses. Sequence alignments indicated that, compared to H3 IAV, these LPAIV strains contained a “N165” glycosite that was four residues further toward the C-terminus and down its resident beta sheet, making it more solvent-accessible (Parsons et al., 2017; Parsons et al., 2020), and glycoproteomics analysis revealed that all the LPAIV strains tested contained complex glycans at the glycosite and did not bind to SP-D significantly compared to H3 HAs. Replacement of the LPAIV glycan at “N165” in H6 recombinant HA with a high-mannose glycan via growth in HEK293 cells under conditions including swainsonine, a potent mannosidase inhibitor, evoked an increase in SP-D preference, thus demonstrating that, the high-mannose glycan, and not strictly the position in the HA tertiary structure, was responsible for the interaction with SP-D in those group 1 AIVs. Essentially, the position of the glycosite in group 1 and group 2 HA appeared to dictate the access of Golgi processing enzymes that either allow elaborate modification of high-mannose glycan to result in complex ones (group 1) or prevent high-mannose glycan processing to complex ones via poor access to ER and Golgi processing machinery.

Sequence alignment of the group 2 LPAIV H4 HAs studied here with H3 HAs produced a different pattern from the group 1 HAs. Clearly, the Q226L/G228S mutations present a possible pandemic risk. Again, our question was would such pandemic strains be susceptible to SP-D? Susceptibility to SP-D would decrease the potential pathogenicity, whereas lower susceptibility to the surfactant would increase potential pathogenicity. Sequence analysis and inspection of available X-ray crystal structures revealed that the position of the H4 HA glycosite N165 was highly similar to that of H3, being high on its resident beta sheet in essentially the same position as that of H3 HAs (Supplementary Figure S1). However, key residues involved in intra-subunit 220 loop contacts between key amino acid residues and the glycan core chitobiose (GlcNAcβ1-4GlcNAc-) moiety differed. A major interaction observed in the H3 structure involved multiple contacts with W222, P221, and S219. The duck H4 HA had leucine instead of tryptophan at position 222, although S219 was present. Subsequently, the orientation of the duck H4 glycan core differed from that of H3. Again, we note that duck contains Q226 and G228, and thus, the avian receptor type. The swine H4 HA, containing L226 and S228, and thus, the human receptor type, contained the H3-like S219 and W222 but did not form close contacts with P221 and was not planar to W222 as in the H3 HA. Neither the swine nor the duck X-ray structures exhibit the more complete glycoform observed in the H3 structure, hinting at more mobility in the H4 duck and swine HAs *versus* H3 HAs. Although the teal10 and teal83 HAs had no X-ray crystal structures available, they were highly similar to duck in the N165 glycosite region except for the presence of W222 in place of L222.

Despite the differences in the crystal structures, the HA glycosite N165 of all of the H4 HAs examined by glycoproteomics analysis herein contained exclusively high-mannose glycan substitutions. Therefore, if such viruses were to infect humans, they would likely be acted upon significantly by SP-D. SP-D surface plasmon resonance studies performed here demonstrated SP-D preference that was similar to that of a seasonal H3N2 HA and much higher than that of H6N2, which contains a complex glycan at N165.

There is clear evidence that SP-D is a key factor in removing influenza virus from the respiratory tissues. It is well documented that the affinity of SP-D for influenza HA is related to key *N-*glycosites in the globular head region of HA (LeVine et al., 2001; Hartshorn et al., 2002; Hartshorn et al., 2008; Tecle et al., 2008; White et al., 2010) and that increasing head high-mannose glycosites enhances this interaction (An et al., 2015; Parsons et al., 2020). This has been previously shown for H3 and H1 viruses. In studies of H3 HA, it has been shown that the absence of the key N165 glycosite enhances pathogenicity in mice (Hawgood et al., 2004). A study that tested historically relevant seasonal influenza-like strains with increasing high-mannose glycans demonstrated decreased pathology in murine lung after exposure. Exposure of SP-D −/− mice to these strains restored susceptibility to infection and increased pathology in respiratory tissues (Wanzeck et al., 2011; An et al., 2015). At the molecular level, based on crystallographic studies, specific mannose residues interact with the SP-D-binding site, the most strongly being the Manα1,2Man-disaccharides that are present in the larger high-mannose glycoforms (Crouch et al., 2009), and the majority of the high-mannose glycans detected at N165 in all four H4 viruses studies here contained these residues. Docking experiments using both H3 and H1 viruses and human wild-type and lower-affinity mutant constructs of SP-D have refined the understanding of protein interaction of glycosite high-mannose glycan interactions with the SP-D carbohydrate recognition domain (Crouch et al., 2011b).

There are a range of clinical implications related to SP-D activities as revealed in group 2 IAV strains lacking N165, certain co-morbidity states, murine models, and SP-D polymorphisms. SP-D polymorphisms have been associated with respiratory infection risk. Those that result in reduced forms of higher-molecular weight multimers have reduced anti-IAV activities and have been shown to increase the risk of respiratory infection in children with associated haplotypes (Thomas et al., 2009). Complications of diabetes, including primary effects of high glucose levels, have been shown to impair SP-D binding in a murine “metabolic syndrome” model, which correlates with the increased risk of respiratory infection in diabetes mellitus (Reading et al., 1998). IAV strains lacking high-mannose glycan at N165 or other high-mannose sites in the globular head have been shown to have decreased activity of SP-D and higher morbidity and mortality in murine models (Hartley et al., 1997; Reading et al., 2009). H5 IAVs, which do not contain high-mannose glycan on the globular head region, are associated with high morbidity and mortality in human outbreaks (Lee et al., 2005; Chen et al., 2006) and a contributing factor may be the inability of SP-D to interact with these strains. Also, previous publications showed that deficiency in SP-D through genetic knockout leads to more severe infections with H3 IAV in murine studies (Vigerust et al., 2007). Recombinant multimeric SP-D has been proposed as a therapeutic intervention (White et al., 2001; Orgeig et al., 2010), which could be useful agents against pandemic and seasonal IAVs that are predicted to contain high-mannose glycans on the HA head region.

In conclusion, the H4 viruses have a demonstrated propensity for the gain of function mutations at key residues in the receptor-binding domain. Adaptation to the swine intermediary host by G228S and to human receptor-binding patterns of the Q226L and G228S adaptations has been documented. However, glycosylation sites N165 in the H4 AIV have a strong propensity to contain high-mannose glycans. These AIVs are susceptible to SP-D activity. This mosaic of RBS changes and head glycosite characteristics may predict key characteristics of infection if these HAs appeared in human infection. That is, human infection may occur based on RBD specificity, but SP-D could be effective for the removal of these AIVs from respiratory tissue.

## Data Availability

The original contributions presented in the study are included in the article/Supplementary Material; further inquiries can be directed to the corresponding author.
