# Supplementary material for: Glycosylation of H4 influenza strains with pandemic potential and susceptibilities to lung surfactant SP-D
Source: Front Mol Biosci. 2023 Jun 13;10:1207670. doi: 10.3389/fmolb.2023.1207670 (PMC10296771; doi:10.3389/fmolb.2023.1207670)
Supplement: Supplementary file 1 [file Table1.DOCX]

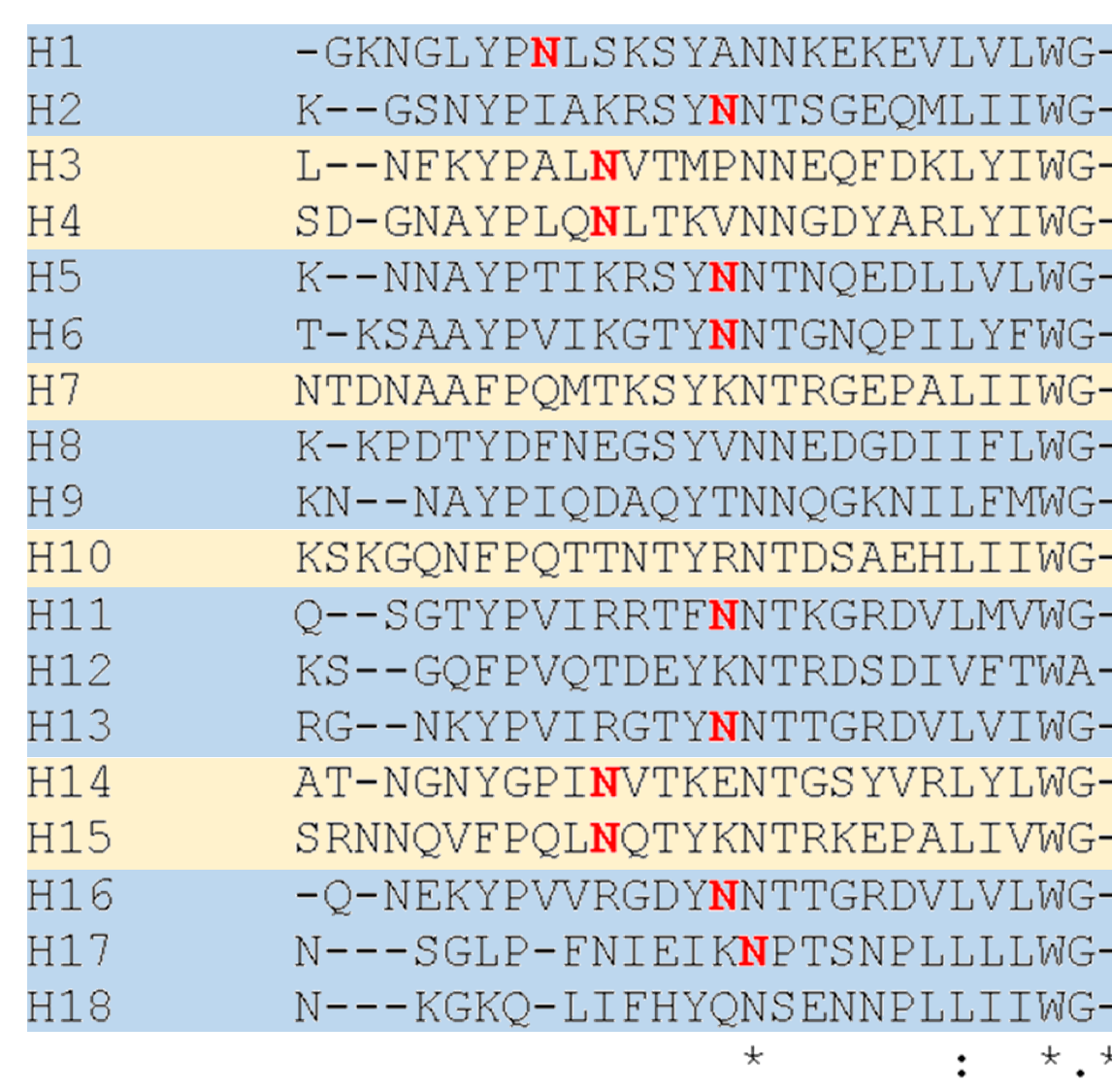


**Supplementary Figure S1**. Alignment of consensus sequences for each HA subtype in the region of the “N165” glycosylation site (red). Group 1 sequences are in blue and Group 2 in yellow. Consensus sequences calculated from alignment of non-redundant sequences for all species for each subtype from the influenza database except H3 which is based on the human sequences only due to the large number of sequences.

duck QNYTGNPVICMGHHAVANGTMVKTLADDQVEVVTAQELVESQNLPELCPSP-LRLVDGQT 59

swine .*........L.....P*.......T...I............H................. 59

teal83 .*........L.....S*.......T..K.............H................. 59

teal2010 .*........L.....S*.......T................H................. 59

duck CDIINGALGSPGCDHLN-GAEWDVFIERPNAV-DTCYPFDVPEYQSLRSILANNGKFEFI 117

swine ...V.........................T............D................. 117

teal83 ...V.........................T............D................. 117

teal2010 ...V......................................D................. 117

“**N165”**

duck A--EEFQWNTV-KQNGKSGACKRANVNDFFNRLNWLVKSDGNAYPLQNLTKINNGDYARL 174

swine ....................................T..........*...V........ 174

teal83 ....................................T..........*...V........ 174

teal2010 ....................................T..........*...V........ 174

**220 loop**

duck YIWGVHHPSTDTEQTNLYKNNPGGVTVSTKTSQTSVVPNIGSRPLVRGQSGRVSFYWTIV 234

swine .......................R.....Q..............W...L.S.I....... 234

teal83 .......................R.....Q..............W.......I....... 234

teal2010 .......................R....................W.......I....... 234

duck EPGDLIVFNTIGNLIAPRGHYKLNNQKKSTILNTAIPIGSCVSKCHTDKGSLSTTKPFQN 294

swine ........................S..........V............R..IT......* 294

teal83 ........................S..........V............R..I.......* 294

teal2010 ........................S.......................R..I.......* 294

duck ISRIAVGDCPRYVKQGSLKLATGMRNIPEKASRGLFGAIAGFIENGWQGLIDGWYGFRHQ 354

swine ....SI....K....................T............................ 354

teal83 ....SI....K....................T............................ 354

teal2010 ....SI....K....................T............................ 354

duck NAEGTGTAADLKSTQAAIDQINGKLNRLIEKTNDKYHQIEKEFEQVEGRIQDLEKYVEDT 414

swine ...............T.................E.......................... 414

teal83 .................................E.......................... 414

teal2010 .................................E.......................... 414

duck KIDLWSYNAELLVALENQHTIDVTDSEMNKLFERVRRQLRENAEDKGNGCFEIFHKCDNN 474

swine .......................................................Q.... 474

teal83 .......................................................Q.... 474

teal2010 .......................................................Q...S 474

duck CIESIRNGTYDHDIYRDEAINNRFQIQGVKLTQGYKEIILWISFSISCFLLVALLLAFIL 534

swine ......*.............................D....................... 534

teal83 ......*..................................................... 534

teal2010 ......*.............................D....................... 534

duck WACQNGNIRCQICI 548

swine .............. 548

teal83 .............. 548

teal2010 .............. 548

**Supplementary Figure S2**. Sequence alignment of H4 HAs studied. Green and asterisks mark potential glycosylation sites. Gray marks the 220 loop with the tryptophan in cyan. Yellow marks residues L226 and S228 that show a preference for human Sialylα2,6 receptors. Alignment by Clustal Omega. For the swine and teal sequences, only residues that differ from the duck sequence are shown.


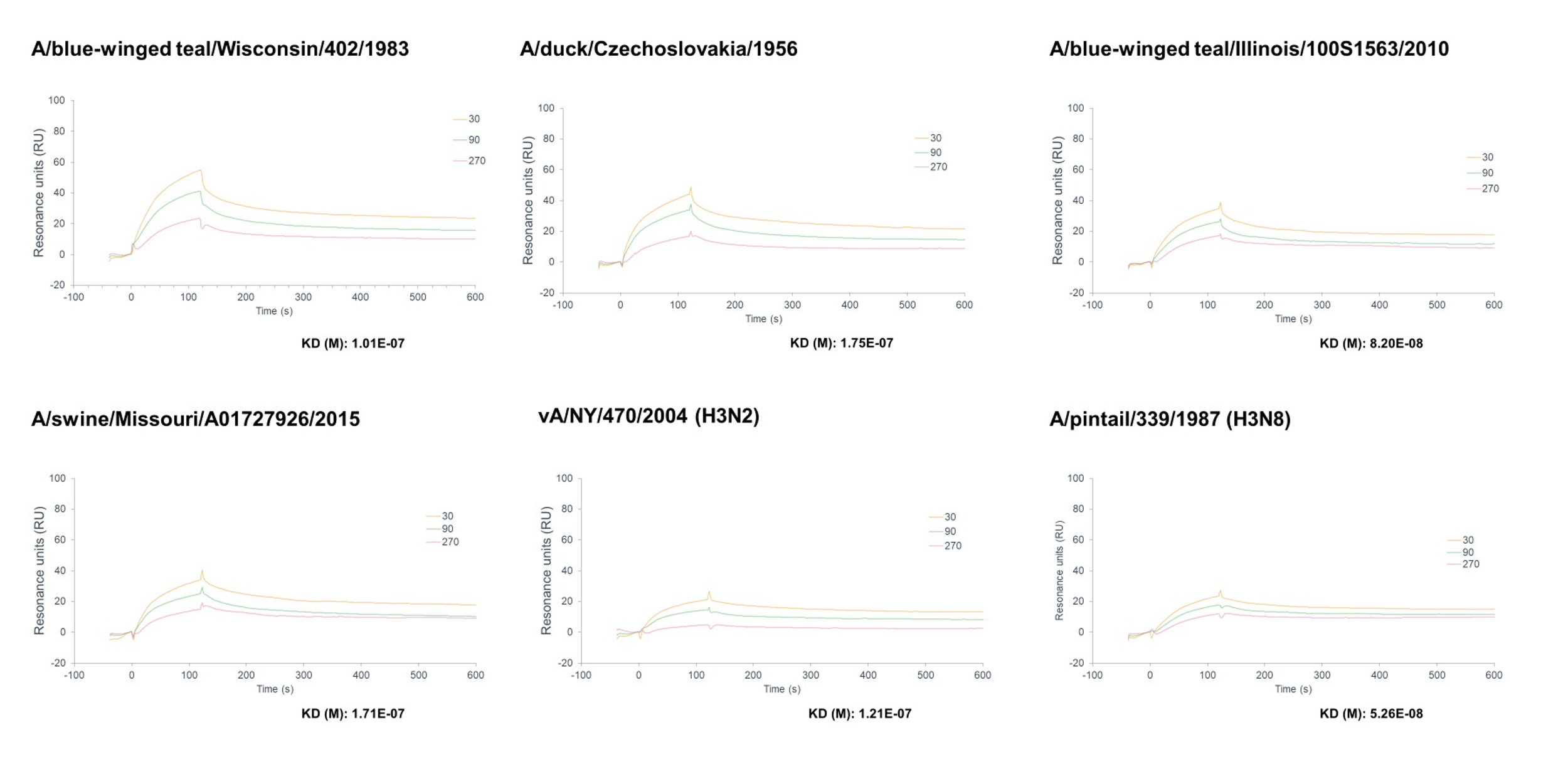


**Supplementary Figure 3**. Surface plasmon resonance analysis of IAV versus SP-D. Biotinylated SP-D (rhSP-D) (dodecamer) coupled to NLC sensor chips was subjected to serial dilutions of H4 T83 (A/Bwteal/Wisconsin/402/83), Duck (A/duck/Czechoslovakia/1956), T10 (A/Bwteal/IL/100S/1563/2010), Swine (A/Swine/Mo/A017/27926/15) and control viruses vA/Ny/470/2004 (H3N2): positive control, A/pintail/339/1987 (H3N8): positive control, and A/mallard/Ohio/249/1998 (H6N1): negative control. The negative control demonstrated no binding affinity (not shown). All tested strains demonstrated similar binding kinetics with KD in simsilar range consistent with H4 viruses having similar binding affinities as H3 viruses.

***
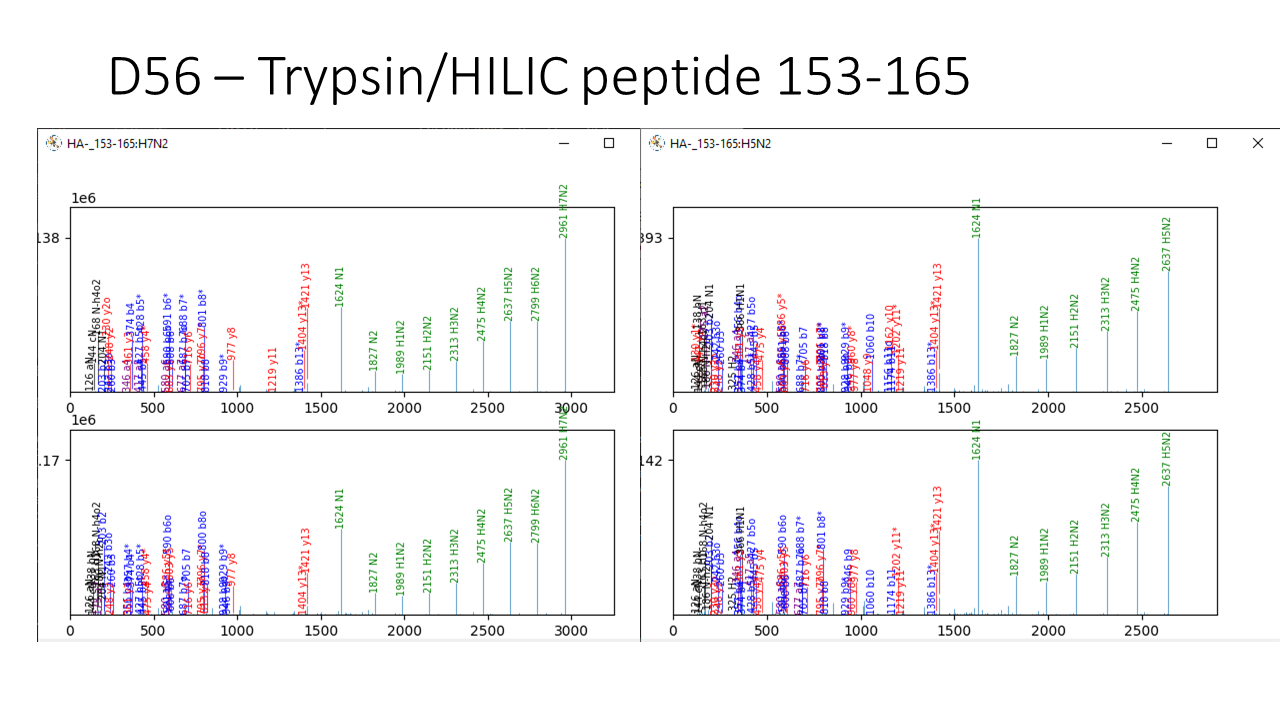
***

**Supplementary Figure 4.** Representative MS^E^ spectra from Duck (A/duck/Czechoslovakia/1956 (H4N6)) HA glycopeptide 153-165 containing the N165 glycosylation site. The glycan moiety on palettes to the left are Hex7HexNAc2 and those to the right are Hex5HexNAc2 consistent with high mannose glycan.

***
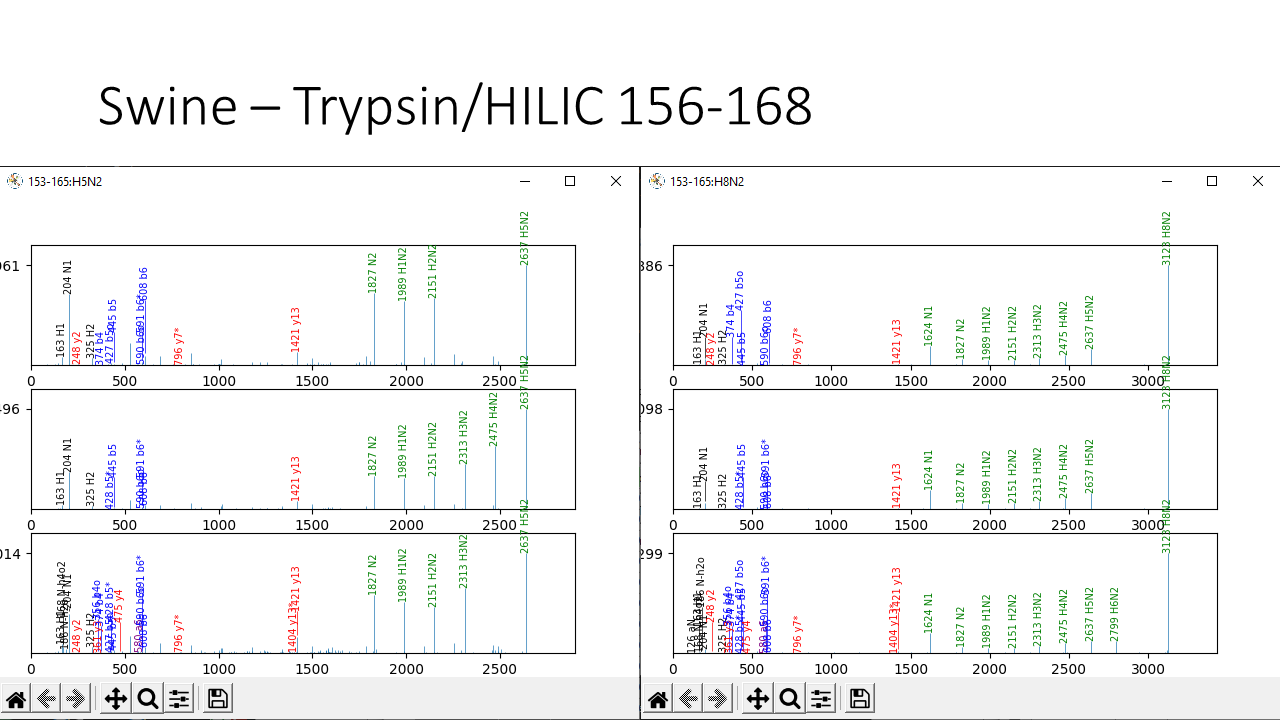
***

**Supplementary Figure 5.** Representative MS^E^ spectra from Swine (A/swine/Missouri/A01727926/2015 (H4N6)) HA glycopeptide 156-168 containing the N165 glycosylation site. The glycan moiety on palettes to the left are Hex5HexNAc2 and those to the right are Hex8HexNAc2 consistent with high mannose glycan.

***
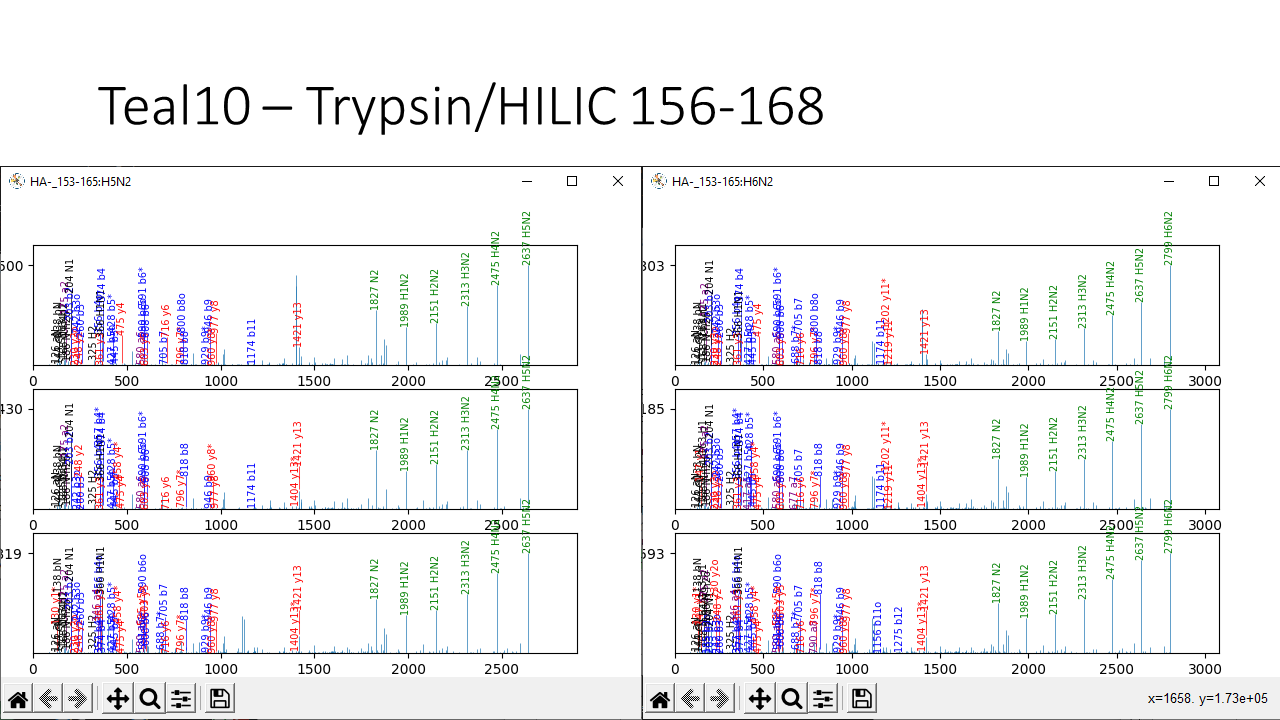
***

**Supplementary Figure 6.** Representative MS^E^ spectra from Teal10 (A/blue-winged teal/Illinois/10OS1563/2010 (H4N6)) HA glycopeptide 156-168 containing the N165 glycosylation site. The glycan moiety on palettes to the left are Hex5HexNAc2 and those to the right are Hex6HexNAc2 consistent with high mannose glycan.

***
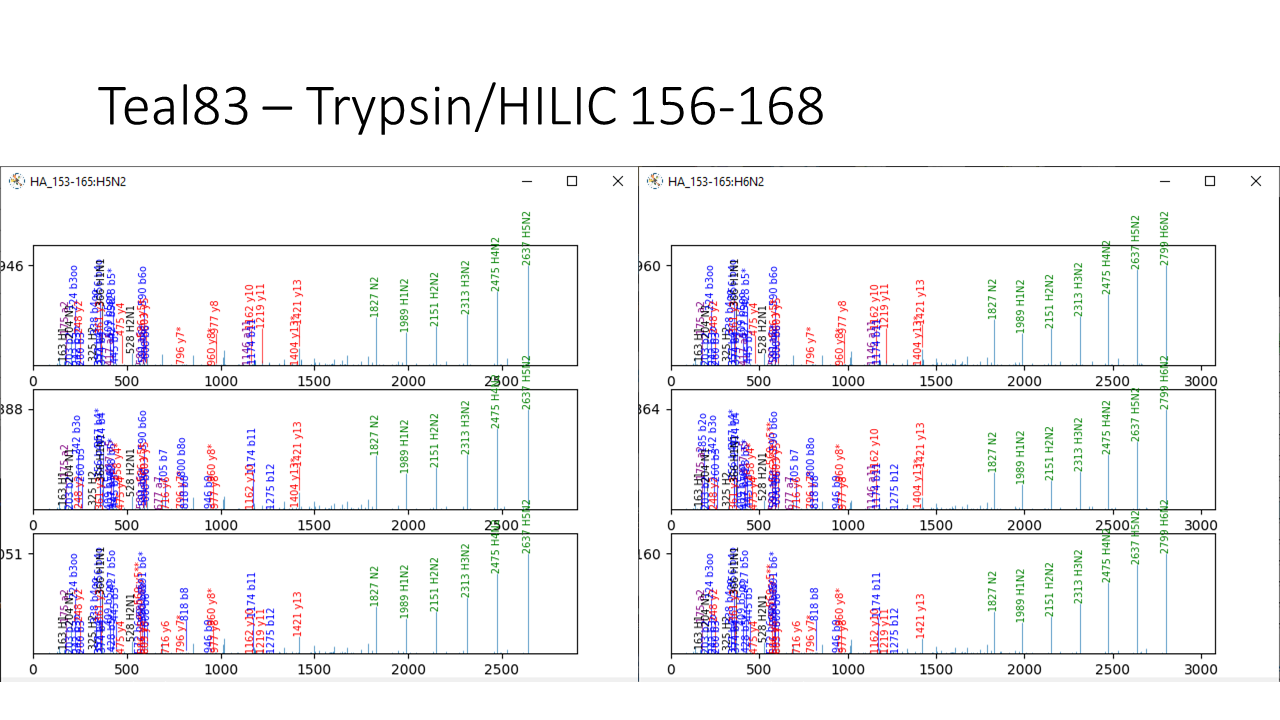
***

Supplementary Figure 7. Representative MS^E^ spectra from Teal83 (A/blue-winged teal/Wisconsin/402/1983 (H4N6)) HA glycopeptide 156-168 containing the N165 glycosylation site. The glycan moiety on palettes to the left are Hex5HexNAc2 and those to the right are Hex6HexNAc2 consistent with high mannose glycan.

**Supplementary Table S1-A. Glycopeptide assignments for egg-grown Teal10**

| Assignments^1^ | **Teal10** | | | |
| --- | --- | --- | --- | --- |
| 153-SDGNAYPLQ**N**LTK-165 | [M+H]^+^ Calc Mass | [M+H]^+^ Mass +/- STD^2^ | Delta^3^ | % intensity +/- STD^4^ |
| Hex3HexNAc2 | 2313.025 | 2313.028 +/- 0.003 | -1.5 | 7.01 +/- 0.84 |
| Hex4HexNAc2 | 2475.077 | 2475.080 +/- 0.001 | -1.1 | 10.12 +/- 0.27 |
| Hex5HexNAc2 | 2637.130 | 2637.134 +/- 0.001 | -1.2 | 10.69 +/- 0.25 |
| Hex6HexNAc2 | 2799.183 | 2799.184 +/- 0.001 | -0.2 | 10.26 +/- 0.07 |
| Hex7HexNAc2 | 2961.236 | 2961.230 +/- 0.002 | 2.0 | 0.09 +/- 0.07 |
| Hex8HexNAc2 | 3123.289 | 3123.278 +/- 0.001 | 3.5 | 61.83 +/- 0.75 |
| 284-GSISTTKPFQ**N**ISR-297 | [M+H]^+^ Calc Mass | [M+H]^+^ Mass +/- STD | Delta | % intensity +/- STD |
| Hex4HexNAc2 | 2590.188 | 2590.187 +/- 0.006 | 0.5 | 5.54 +/- 0.33 |
| Hex5HexNAc2 | 2752.241 | 2752.243 +/- 0.001 | -0.6 | 46.25 +/- 2.08 |
| Hex7HexNAc2 | 3076.347 | 3076.343 +/- .001 | 1.3 | 33.97 +/- 1.33 |
| Hex4HexNAc3 | 2793.268 | 2793.269 +/- .001 | -0.4 | 14.24 +/- 0.44 |
| 481-**N**GTYDHDIYRDEAINNR-497 | [M+H]^+^ Calc Mass | [M+H]^+^ Mass +/- STD | Delta | % intensity +/- STD |
| Hex3HexNAc3 | 3161.329 | 3161.324 +/- 0.002 | 1.9 | 5.97 +/- 0.10 |
| dHex1Hex3HexNAc3 | 3307.387 | 3307.386 +/- 0.000 | 0.5 | 4.50 +/- 0.06 |
| Hex4HexNAc3 | 3323.382 | 3323.381 +/- 0.001 | 0.5 | 14.01 +/- 0.07 |
| dHex1Hex4HexNAc3 | 3469.440 | 3469.437 +/- 0.000 | 0.9 | 12.42 +/- 0.10 |
| Hex5HexNAc4 | 3688.514 | 3688.510 +/- 0.001 | 1.2 | 19.56 +/- 0.14 |
| dHex1Hex5HexNAc4 | 3834.572 | 3834.566 +/- 0.001 | 1.6 | 26.95 +/- 0.27 |
| dHex2Hex5HexNAc4 | 3980.630 | 3980.621 +/- 0.001 | 2.3 | 9.80 +/- 0.06 |
| dHex1Hex5HexNAc5 | 4037.652 | 4037.635 +/- 0.001 | 4.2 | 6.78 +/- 0.08 |

^1^Glycans found associated with each peptide are listed beneath the peptide

^2^Average measured mass +/- one standard deviation based on assignments found at same retention times across three injections

^3^Delta = (calculated mass – average found mass) in ppm

^4^Percent intensity calculated per peptide.

**Supplementary Table S1-B. Glycopeptide assignments for egg-grown Teal83**

| Assignments | **Teal83** | | | |
| --- | --- | --- | --- | --- |
| 153-SDGNAYPLQ**N**LTK-165 | [M+H]^+^ Calc Mass | [M+H]^+^ Mass +/- STD | Delta | % intensity +/- STD |
| Hex3HexNAc2 | 2313.025 | 2313.032 +/- 0.002 | -3.1 | 6.29 +/- 0.66 |
| Hex4HexNAc2 | 2475.077 | 2475.084 +/- 0.001 | -2.8 | 7.31 +/- 0.78 |
| Hex5HexNAc2 | 2637.130 | 2637.135 +/- 0.001 | -1.9 | 11.40 +/- 1.50 |
| Hex6HexNAc2 | 2799.183 | 2799.185 +/- 0.002 | -0.5 | 10.61+/- 0.65 |
| Hex8HexNAc2 | 3123.289 | 3123.279 +/- 0.002 | 3.0 | 64.39 +/- 3.58 |
| 284-GSISTTKPFQ**N**ISR-297 | [M+H]^+^ Calc Mass | [M+H]^+^ Mass +/- STD | Delta | % intensity +/- STD |
| Hex3HexNAc2 | 2428.135 | 2428.141 +/- 0.002 | -2.3 | 12.81 +/- 0.53 |
| Hex4HexNAc2 | 2590.188 | 2590.185 +/- 0.002 | 1.4 | 16.82 +/- 0.29 |
| Hex5HexNAc2 | 2752.241 | 2752.239 +/- 0.003 | 0.7 | 26.73 +/- 3.06 |
| Hex7HexNAc2 | 3076.347 | 3076.345+/- 0.001 | 0.6 | 43.64 +/- 2.74 |
| 481-**N**GTYDHDIYRDEAINNR-497 | [M+H]^+^ Calc Mass | [M+H]^+^ Mass +/- STD | Delta | % intensity +/- STD |
| Hex3HexNAc3 | 3161.329 | 3161.328 +/- 0.002 | 0.5 | 6.37 +/- 0.29 |
| dHex1Hex3HexNAc3 | 3307.387 | 3307.386 +/- 0.001 | 0.5 | 3.89 +/- 0.19 |
| Hex4HexNAc3 | 3323.382 | 3323.382 +/- 0.002 | 0.0 | 15.05 +/- 0.52 |
| dHex1Hex4HexNAc3 | 3469.440 | 3469.441 +/- 0.002 | 0.0 | 11.07 +/- 0.20 |
| Hex4HexNAc4 | 3526.462 | 3526.454 +/- 0.004 | 2.2 | 4.00 +/- 0.42 |
| dHex1Hex4HexNAc4 | 3672.520 | 3672.516 +/- 0.002 | 1.0 | 4.89 +/- 0.20 |
| Hex5HexNAc4 | 3688.515 | 3688.513 +/- 0.002 | 0.4 | 22.17 +/- 0.79 |
| dHex1Hex5HexNAc4 | 3834.572 | 3834.570 +/- 0.001 | 0.7 | 26.16 +/- 0.18 |
| dHex1Hex5HexNAc5 | 4037.652 | 4037.640 +/- 0.003 | 2.8 | 6.41 +/- 0.18 |

**Supplementary Table S1-C. Glycopeptide assignments for egg-grown duck**

| Assignments | **Duck** | | | |
| --- | --- | --- | --- | --- |
| 153-SDGNAYPLQ**N**LTK-165 | [M+H]^+^ Calc Mass | [M+H]^+^ Mass +/- STD | Delta | % intensity +/- STD |
| Hex3HexNAc2 | 2313.025 | 2313.029 +/- 0.000 | -1.7 | 7.31 +/- 1.41 |
| Hex4HexNAc2 | 2475.077 | 2475.081 +/- 0.000 | -1.3 | 7.43 +/- 1.88 |
| Hex5HexNAc2 | 2637.130 | 2637.132 +/- 0.000 | -0.5 | 16.59 +/- 1.93 |
| Hex7HexNAc2 | 2961.236 | 2961.229 +/- 0.001 | 2.3 | 30.15 +/- 4.58 |
| Hex8HexNAc2 | 3123.289 | 3123.274 +/- 0.001 | 4.6 | 44.05 +/- 6.60 |
| 284-GSLSTTKPFQ**N**ISR-297 | [M+H]^+^ Calc Mass | [M+H]^+^ Mass +/- STD | Delta | % intensity +/- STD |
| Hex3HexNAc2 | 2428.135 | 2428.140 +/- 0.001 | -1.6 | 7.25 +/- 2.24 |
| Hex5HexNAc2 | 2752.241 | 2752.241 +/- 0.002 | 0.2 | 19.15 +/- 0.29 |
| Hex7HexNAc2 | 3076.347 | 3076.338 +/- 0.003 | 2.8 | 16.84 +/- 0.50 |
| Hex4HexNAc3 | 2793.268 | 2793.267 +/- 0.001 | 0.3 | 8.84 +/- 0.81 |
| dHex1Hex4HexNAc3 | 2939.326 | 2939.316 +/- 0.002 | 3.3 | 1.96 +/- 0.81 |
| dHex1Hex5HexNAc3 | 3101.378 | 3101.363 +/- 0.001 | 5.1 | 2.19 +/- 0.05 |
| Hex6HexNAc3 | 3117.373 | 3117.365 +/- 0.002 | 2.8 | 14.02 +/- 0.41 |
| Hex4HexNAc4 | 2996.347 | 2996.337 +/- 0.002 | 3.3 | 2.47 +/- 0.66 |
| Hex5HexNAc4 | 3158.400 | 3158.395 +/- 0.002 | 1.5 | 20.19 +/- 1.24 |
| Hex5HexNAc5 | 3361.479 | 3361.473 +/- 0.003 | 2.0 | 7.84 +/- 0.05 |
| 481-**N**GTYDHDIYRDEAINNR-497 | [M+H]^+^ Calc Mass | [M+H]^+^ Mass +/- STD | Delta | % intensity +/- STD |
| Hex3HexNAc3 | 3161.329 | 3161.318 +/- 0.003 | 3.5 | 5.51 +/- 1.20 |
| dHex1Hex3HexNAc3 | 3307.387 | 3307.380 +/- 0.001 | 2.4 | 4.06 +/- 0.98 |
| Hex4HexNAc3 | 3323.382 | 3323.374 +/- 0.002 | 2.4 | 17.34 +/- 1.41 |
| dHex1Hex4HexNAc3 | 3469.440 | 3469.433 +/- 0.003 | 2.0 | 13.32 +/- 1.40 |
| Hex4HexNAc4 | 3526.462 | 3526.453 +/- 0.002 | 2.5 | 3.08 +/- 1.27 |
| dHex1Hex4HexNAc4 | 3672.520 | 3672.509 +/- 0.002 | 3.0 | 4.00 +/- 1.65 |
| Hex5HexNAc4 | 3688.515 | 3688.503 +/- 0.002 | 3.0 | 18.79 +/- 4.96 |
| dHex1Hex5HexNAc4 | 3834.572 | 3834.561 +/- 0.001 | 2.9 | 24.56 +/- 1.49 |
| dHex2Hex5HexNAc4 | 3980.630 | 3980.614 +/- 0.003 | 4.0 | 7.06 +/- 0.95 |
| dHex1Hex5HexNAc5 | 4037.652 | 4037.631 +/- 0.003 | 5.0 | 5.67 +/- 1.39 |

**Supplementary Table S1-D. Glycopeptide assignments for egg-grown swine**

| Assignments | | **Swine** | | | |
| --- | --- | --- | --- | --- | --- |
| 156-SDGNAYPLQ**N**LTK-168 | | [M+H]^+^ Calc Mass | [M+H]^+^ Mass +/- STD | Delta | % intensity +/- STD |
| Hex3HexNAc2 | | 2313.025 | 2313.015 +/- 0.015 | 4.2 | 3.75 +/- 0.28 |
| Hex4HexNAc2 | | 2475.077 | 2475.064 +/- 0.021 | 5.4 | 5.14 +/- 0.69 |
| Hex5HexNAc2 | | 2637.130 | 2637.125 +/- 0.007 | 2.1 | 5.48 +/- 2.18 |
| Hex8HexNAc2 | | 3123.289 | 3123.294 +/- 0.008 | 0.1 | 85.62 +/- 2.57 |
| 284-GSITTTKPFQ**N**ISR-297 | | [M+H]^+^ Calc Mass | [M+H]^+^ Mass +/- STD | Delta | % intensity +/- STD |
| Hex3HexNAc2 | | 2442.151 | 2442.165 +/- 0.028 | -5.8 | 4.75 +/- 1.25 |
| Hex4HexNAc2 | | 2604.204 | 2604.216 +/- 0.022 | -4.4 | 6.96 +/- 1.23 |
| Hex5HexNAc2 | | 2766.257 | 2766.271 +/- 0.024 | -8.0 | 18.74 +/- 7.43 |
| Hex6HexNAc2 | | 2928.310 | 2928.330 +/- 0.028 | -6.9 | 12.32 +/- 1.67 |
| Hex7HexNAc2 | | 3090.363 | 3090.386 +/- 0.031 | -7.4 | 22.25 +/- 4.08 |
| Hex4HexNAc3 | | 2807.283 | 2807.295 +/- 0.022 | -4.1 | 4.25 +/- 3.21 |
| Hex6HexNAc3 | | 3131.389 | 3131.411 +/- 0.030 | -7.1 | 10.70 +/- 1.32 |
| dHex1Hex6HexNAc3 | | 3277.447 | 3277.476 +/- 0.040 | -8.8 | 6.10 +/- 1.32 |
| Hex5HexNAc4 | | 3172.416 | 3172.439 +/- 0.030 | -7.2 | 13.93 +/- 1.70 |
| 481-**N**GTYDHDIYRDEAINNR-497 | | [M+H]^+^ Calc Mass | [M+H]^+^ Mass +/- STD | Delta | % intensity +/- STD |
| Hex3HexNAc3 | | 3161.329 | 3161.375 +/- 0.027 | -14.4 | 4.37 +/- 3.79 |
| Hex4HexNAc3 | | 3323.382 | 3323.410 +/- 0.035 | -8.4 | 13.28 +/- 2.68 |
| dHex1Hex4HexNAc3 | | 3469.440 | 3469.469 +/- 0.037 | -8.3 | 11.27 +/- 1.84 |
| Hex4HexNAc4 | | 3526.462 | 3526.512 +/- 0.034 | -14.2 | 2.98 +/- 2.62 |
| Hex5HexNAc4 | | 3688.515 | 3688.549 +/- 0.039 | -9.4 | 28.38 +/- 3.43 |
| dHex1Hex5HexNAc4 | | 3834.572 | 3834.609 +/- 0.038 | -9.6 | 30.66 +/- 8.27 |
| dHex2Hex5HexNAc4 | | 3980.630 | 3980.683 +/- 0.031 | -13.3 | 5.14 +/- 4.48 |
| dHex1Hex5HexNAc5 | | 4037.652 | 4037.712 +/- 0.035 | -14.8 | 3.92 +/- 3.41 |
| NeuAc3Hex7HexNAc6 | 4479.687 | | 4479.674 +/- 0.006 | 3.0 | 0.46 +/- 0.04 |
| NeuAc1dHex1Hex7HexNAc6 | 4043.555 | | 4043.564 +/- 0.006 | -2.3 | 0.36 +/- 0.03 |
| NeuAc2dHex1Hex7HexNAc6 | 4334.650 | | 4334.647 +/- 0.002 | 0.7 | 2.50 +/- 0.15 |
| NeuAc3dHex1Hex7HexNAc6 | 4625.745 | | 4625.780 +/- 0.003 | -7.3 | 10.53 +/- 0.70 |
| NeuAc4dHex1Hex7HexNAc6 | 4916.841 | | 4916.823 +/- 0.003 | 3.7 | 9.45 +/- 0.58 |
| NeuAc3dHex2Hex7HexNAc6 | 4771.803 | | 4771.796 +/- 0.003 | 1.6 | 0.41 +/- 0.03 |
| dHex1Hex7HexNAc7 | 3955.539 | | 3955.538 +/- 0.002 | 0.2 | 4.81 +/- 0.80 |
| NeuAc2dHex1Hex7HexNAc7 | 4537.729 | | 4537.726 +/- 0.001 | 0.8 | 1.39 +/- 0.09 |
| NeuAc3dHex1Hex7HexNAc7 | 4828.825 | | 4828.809 +/- 0.004 | 3.2 | 3.51 +/- 0.30 |
| NeuAc4dHex1Hex7HexNAc7 | 5119.920 | | 5119.889 +/- 0.002 | 6.1 | 3.04 +/- 0.18 |

^1^ Assignments for peptide 12-23 were from trypsin/chymotrypsin cleaved peptides. All others were from trypsin only.

**Supplementary Table S2. Close contacts seen in Figure 1.**

| **Close contacts between** | | |
| --- | --- | --- |
| **Set 1**: 5xL1, subunit A, peptide | | |
| **Set 2**: 5xL1, subunit C, sugar at N165(2) [NAG601] | | |
| **Atom in set 1^a^** | **Atom in set 2^b^** | **Distance** |
| A/219(SER)/OG | C/601(NAG)/C5 | 3.66 |
| A/219(SER)/OG | C/601(NAG)/C6 | 3.22 |
| A/219(SER)/OG | C/601(NAG)/O5 | 3 |
| A/219(SER)/CB | C/601(NAG)/O5 | 3.77 |
| **A/219(SER)/OG^c^** | **C/601(NAG)/O6** | **2.87** |
| A/186(SER)/OG | C/601(NAG)/O6 | 3.97 |
| A/187(THR)/CG2 | C/601(NAG)/O6 | 3.58 |
|  | | |
| **Close contacts between** | | |
| **Set 1**: 5xL2, subunit A, peptide | | |
| **Set 2**: 5xL2, subunit C, sugar at N165(2) [NAG601] | | |
| **Atom in set 1^a^** | **Atom in set 2** | **Distance** |
| A/219(SER)/CB | C/601(NAG)/C1 | 3.8 |
| A/222(TRP)/NE1 | C/601(NAG)/O4 | 3.39 |
| A/222(TRP)/CE2 | C/601(NAG)/O4 | 3.5 |
| A/222(TRP)/CZ2 | C/601(NAG)/O4 | 3.54 |
|  | | |
| **Close contacts between** | | |
| **Set 1**: 4FNK, subunit C, peptide | | |
| **Set 2**: 4FNK, subunit A, sugar at N165 [NAG404] | | |
| **Atom in set 1** | **Atom in set 2** | **Distance** |
| C/219(SER)/CB | A/404(NAG)/C1 | 3.95 |
| C/219(SER)/OG | A/404(NAG)/C1 | 3.87 |
| C/219(SER)/OG | A/404(NAG)/C2 | 3.87 |
| C/219(SER)/OG | A/404(NAG)/N2 | 3.13 |
| C/222(TRP)/N | A/405(NAG)/C7 | 3.96 |
| C/222(TRP)/CB | A/405(NAG)/O3 | 3.74 |
| C/222(TRP)/CG | A/405(NAG)/O3 | 3.87 |
| C/222(TRP)/CE3 | A/405(NAG)/O5 | 3.96 |
| C/222(TRP)/CZ3 | A/405(NAG)/O6 | 3.12 |
| C/222(TRP)/CH2 | A/405(NAG)/O6 | 2.97 |
| C/222(TRP)/CE2 | A/405(NAG)/O6 | 3.91 |
| C/222(TRP)/CE3 | A/405(NAG)/O6 | 3.67 |
| C/222(TRP)/CZ2 | A/405(NAG)/O6 | 3.41 |
| C/222(TRP)/CB | A/405(NAG)/O7 | 3.6 |
| C/221(PRO)/CA | A/405(NAG)/O7 | 3.35 |
| C/221(PRO)/C | A/405(NAG)/O7 | 3.59 |
| **C/222(TRP)/N** | **A/405(NAG)/O7** | **2.85** |
| C/222(TRP)/CA | A/405(NAG)/O7 | 3.85 |
| C/222(TRP)/CD1 | A/406(BMA)/C5 | 3.98 |
| C/222(TRP)/NE1 | A/406(BMA)/C5 | 3.95 |

^a^ Numbering adjusted to match that for H3

^b^ NAG=N-acetyl-glucosamine, BMA= beta-D-mannose

^c^ Bold contacts are hydrogen bonds

**Supplemental Table S3. SPR Analysis after Digestion with Endo F1**.

| **Sample** | **Biotin-labeled SP-D 4 mg/mL~ Final concentration 5 ug/mL** | | |
| --- | --- | --- | --- |
|  | **endo** | **control** | **% loss** |
|  | **RU** | **RU** |  |
| A/pintail/339/1987 (H3N8) 1:10 | 20.4 | 27.7 | 26.5 |
| A/pintail/339/1987 (H3N8) 1:30 | 7 | 9.2 | 24.1 |
| A/pintail/339/1987 (H3N8) 1:90 | 2.5 | 3.9 | 36.3 |
| A/blue-winged teal/Wiscosin/402/1983 (H4N6) 1:10 | 14.6 | 17.5 | 16.4 |
| A/blue-winged teal/Wiscosin/402/1983 (H4N6) 1:30 | 4.8 | 5.5 | 12.6 |
| A/blue-winged teal/Wiscosin/402/1983 (H4N6) 1:90 | 1.2 | 1.4 | 19.1 |
| A/blue-winged teal/Illinois/100S1563/2010 (H4N6) 1:10 | 11 | 14.9 | 25.8 |
| A/blue-winged teal/Illinois/100S1563/2010 (H4N6) 1:30 | 3.6 | 4.9 | 26 |
| A/blue-winged teal/Illinois/100S1563/2010 (H4N6) 1:90 | 1.1 | 1.8 | 38 |
